# Utilization of Platelet-Rich Plasma for a Fistula With Subcutaneous Cavity Following Septic Bursitis: A Case Report

**Published:** 2015-07-21

**Authors:** Satoshi Kushida, Natsuko Kakudo, Naoki Morimoto, Yudai Mori, Kenji Kusumoto

**Affiliations:** ^a^Department of Plastic and Reconstructive Surgery, Kawachi General Hospital, Yokomakura, Higashi-Osaka, Osaka, Japan; ^b^Department of Plastic and Reconstructive Surgery, Kansai Medical University, Shin-machi, Hirakata, Osaka, Japan

**Keywords:** platelet-rich plasma, wound healing, fistula, subcutaneous cavity, septic bursitis

## Abstract

In platelet-rich plasma (PRP) therapy, various growth factors and cytokines released the α-granules contained in platelets after activation can potentially enhance wound healing by delivering. We report a patient in whom treatment with PRP, prepared using a syringe-centrifugation-system PRP kit (KYOCERA Medical PRP Kit), for a fistula following bursitis of the lateral malleolus, which could not be healed with conventional wound therapy, led to successful healing. A 58-year-old man was on dialysis for type II diabetes and chronic renal failure. In the left lateral malleolus, septic bursitis developed, leading to a refractory fistula with a subcutaneous cavity measuring 4 × 3 cm, which persisted for more than 2 months. Platelet-rich plasma was prepared using the KYOCERA Medical PRP Kit (KYOCERA Medical Corporation, Osaka, Japan) and infused into the cavity twice to close it. After this procedure, the cavity size reduced, but the orifice and subcutaneous cavity were not closed. Therefore, additional PRP therapy was conducted after 10 weeks of the first PRP session. Complete closure was achieved 13 weeks after the first PRP therapy. In the present case, PRP was prepared using the KYOCERA Medical PRP Kit, and wound healing of a fistula with subcutaneous cavity following bursitis of the lateral malleolus was successfully cured. The KYOCERA Medical PRP Kit was useful, because PRP could be prepared simply and inexpensively using the syringe-centrifugation system.

## INTRODUCTION

Platelet-rich plasma (PRP) is a concentrated source of autologous platelets. It contains several different growth factors, such as platelet-derived growth factor (PDGF), transforming growth factor-β (TGF-β), and vascular endothelial growth factor (VEGF), as well as other cytokines that promote the healing of bone and soft tissue.^1^ The KYOCERA Medical PRP Kit (KYOCERA Medical Corporation, Osaka, Japan) facilitates the centrifugation of a blood-containing injector using a centrifuge. We previously reported that high-quality PRP could be prepared simply and inexpensively.^2^ Initially, PRP was primarily used to assist with bone reconstruction in the field of oral and maxillofacial surgery.^3^^,^^4^ However, recently, it has been used for the treatment of refractory diabetic ulcers in the field of plastic surgery,^5^ cosmetic treatment of facial rejuvenation,^6^ and the treatment of Achilles tendon rupture^7^^,^^8^ and ligament injury.

The pathogenesis of bursitis of the lateral malleolus involves infection-, trauma-, or inflammatory articular disease–related inflammation of the joint capsule in an area adjacent to the osseous process of the ankle. The overlaid skin of the bursa is damaged, leading to ulceration or fistula formation in some cases. In patients with underlying diseases such as diabetes, healing is sometimes delayed.

Here, we report a patient in whom treatment with PRP, prepared using a syringe-centrifugation system PRP kit (KYOCERA Medical PRP Kit), of a fistula following bursitis of the lateral malleolus, which could not be cured with conventional wound therapy, led to successful healing.

## METHODS

**Figure 1:**
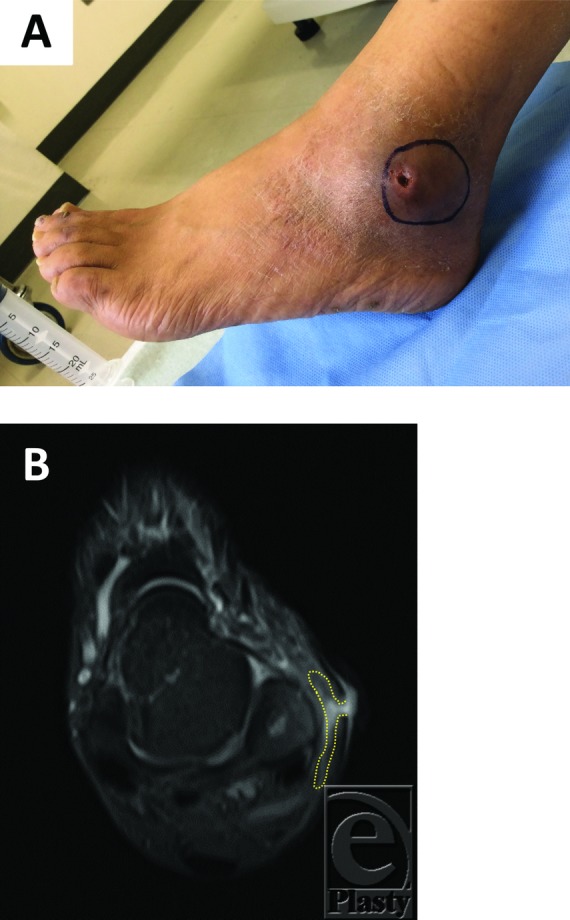
(a) Macroscopic finding showed that a fistula with subcutaneous cavity measuring 4 × 3 cm was observed in the left lateral malleolus. The mark indicates the margin of the subcutaneous cavity. (b) Computed tomographic findings of the left lateral malleolus. The yellow dotted line indicates the margin of the subcutaneous cavity.

**Figure 2:**
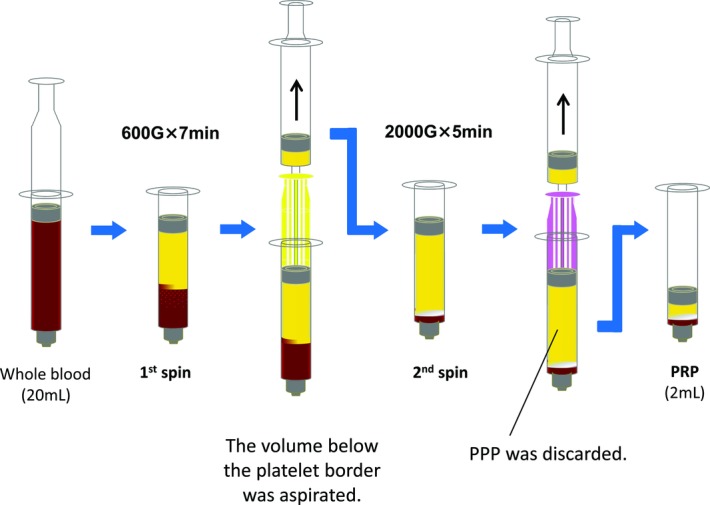
PRP preparation with the KYOCERA Medical PRP Kit (2-session centrifugation method) is shown in the schema. PRP indicates platelet-rich plasma; PPP, platelet poor plasma.

The patient was a 58-year-old man. He consulted our hospital for a refractory subcutaneous cavity following septic bursitis of the left lateral malleolus. His medical history consisted of type II diabetes, chronic renal failure (on dialysis), hypertension, and schizophrenia. For diabetes control, insulin therapy had been undertaken, and the HbA_1c_ level was approximately 7%. The subcutaneous cavity measured 4 × 3 cm (Figs 1*a* and 1*b*). Osteomyelitis was not noted. We applied Gentacin Ointment 0.1% (MSD K.K., Tokyo, Japan) or Prostandin Ointment (Ono Pharmaceutical Co, Ltd, Osaka, Japan) to the wound for 11 weeks before PRP therapy, but there was no healing tendency. Therefore, PRP therapy was indicated. The protocol for this treatment was approved by the Ethics Review Board of Kansai Medical University. Using the KYOCERA Medical PRP Kit (KYOCERA Medical Corporation), 2 mL of PRP was prepared from 20 mL of whole blood according to the manufacturer's instructions.^2^ It was centrifuged twice; the first session was performed at 600 *g* × 7 minutes and the second session at 2000 *g* × 5 minutes. PPP (platelet-poor plasma) was discarded after second centrifugation (Fig 2). The PRP was activated with 0.5 M CaCl_2_ at one-tenth of the amount of the total PRP according to the manufacturer's instructions. Activated PRP was infused into the subcutaneous cavity. After PRP therapy, the wound was washed using physiological saline and covered with petrolatum fabric dressing every 3 days. Subsequently, ointment therapy using Vaseline was continued.

## RESULTS

**Figure 3:**
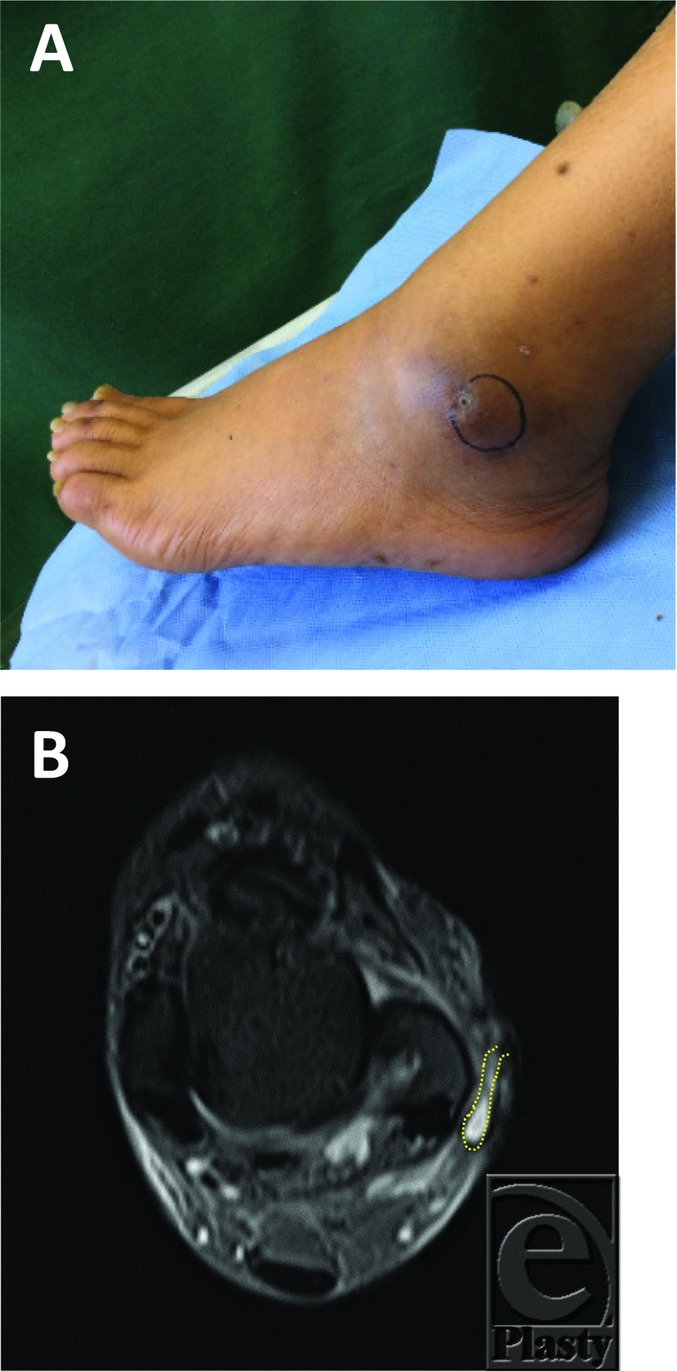
(a) Macroscopic finding showed that the pocket was reduced 10 weeks after the first PRP therapy. (b) Computed tomographic findings of the left lateral malleolus showed that the cavity size reduced after the first PRP session. The yellow dotted line indicates the margin of the subcutaneous cavity. PRP indicates platelet-rich plasma.

**Figure 4:**
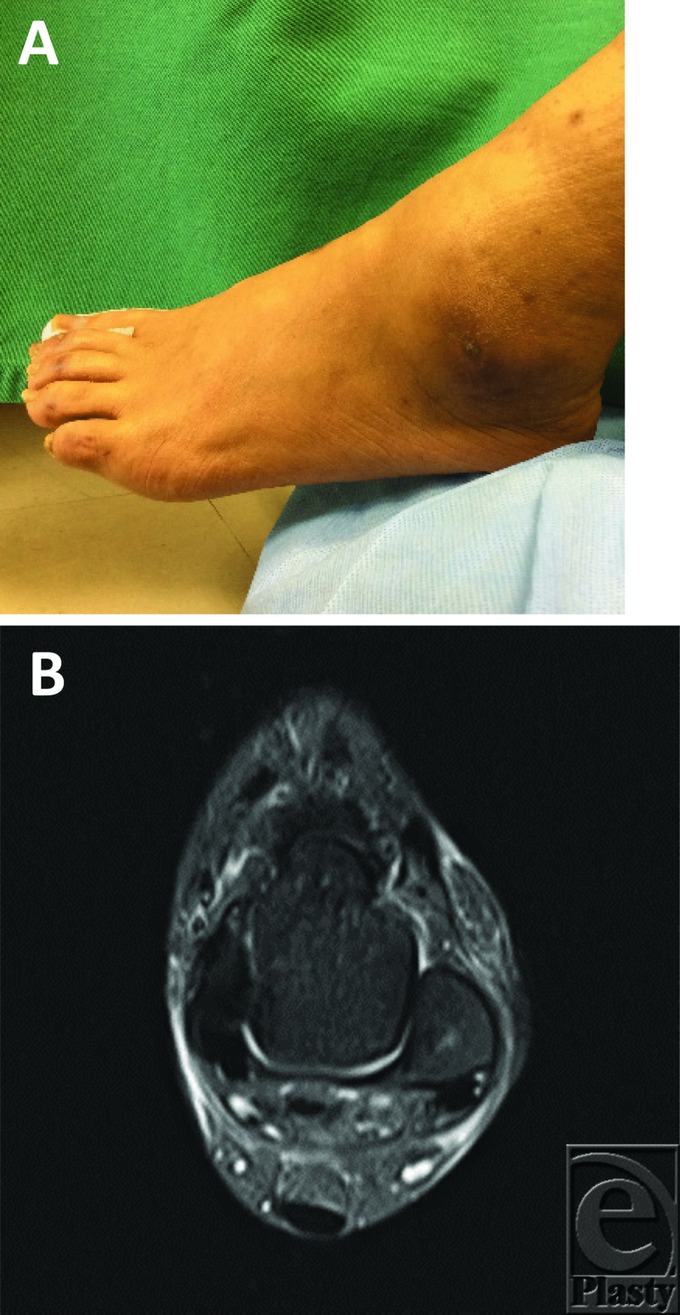
(a) Macroscopic finding showed that the orifice and the cavity were closed completely, and the skin color had improved 13 weeks after the first PRP therapy (3 weeks after the second PRP therapy). (b) Computed tomographic findings of the left lateral malleolus show that the cavity has disappeared.

After the PRP therapy, the cavity size reduced (Figs 3*a* and 3*b*), but the orifice and the subcutaneous cavity were not closed. Therefore, additional PRP therapy was conducted after 10 weeks of the first PRP session. Complete closure was achieved in 13 weeks after the first PRP therapy (3 weeks after the second PRP therapy) (Figs 4*a* and 4*b*).

## DISCUSSION

PRP therapy is a method in which platelets are concentrated from whole blood collected using the standard method to harness the topical actions of autologous cytokines, growth factors/cytokines released from the α-granules of platelets after activation.^1^^,^^9^^,^^10^ Activated PRP contains a large volume of autologous growth factors, such as PDGF, TGF-β, and VEGF, and cytokines that cause cell proliferation/chemotaxis and angiogenesis. Several studies reported that treatment with PRP was useful for tissue regeneration.^2^^,^^11^ The clinical usefulness of PRP for the treatment of refractory ulcers, such as diabetic or pressure ulcers,^5^ bone regeneration/healing,^4^ and facial rejuvenation for facial/hand wrinkles^6^ in the field of plastic and reconstructive, aesthetic surgery, has been emphasized.

In this report, a case report of a patient with a refractory fistula with wide subcutaneous cavity is presented. In general, lateral calcaneal flap is listed as a surgical treatment to cover dermal/subcutaneous defects in the lateral malleolus. However, in the present case, dermal blood flow might be reduced with diabetes and renal dialysis and wound healing may be delayed. Therefore, this may not become the first option. In this case, saline irrigation and conventional wound therapy failed to promote healing, and the orifice and the cavity were not reduced for more than 2 months. Then, PRP therapy was indicated. After the first PRP session, a reduction in the cavity size was observed. Complete closure of the orifice and the cavity was noted 3 weeks after the second PRP session. Several studies reported that PRP promotes dermal angiogenesis and epithelization.^10^^,^^12^ Furthermore, another study indicated that PRP differentiates fibroblasts into myofibroblasts, promoting wound contraction.^13^ A randomized, controlled trial showed that treatment with autologous PRP gel leads to the healing of refractory diabetic foot ulcers.^5^ According to a clinical report, PRP is also effective for other refractory ulcers.^5^^,^^14^ In the present case, these functions may also have intricately led to wound healing.

We propose that the therapy is suitable for wounds with delayed healing with reduced dermal blood flow because of diabetes, wounds having resistance to standard ointment therapy, and clinically controlled local infections. PRP is markedly safe because it is autologous and blood sampling is not invasive, allowing repeated application; it contains several cytokines, for which a synergistic effect can be expected. In addition, PRP therapy can be performed in outpatient clinics without hospitalization.

There are 2 methods to prepare PRP: 1- and 2-session centrifugation methods. Although PRP-preparing kits have been developed by various companies,^2^ the KYOCERA Medical PRP Kit facilitates the preparation of PRP with a semi-closed syringe centrifugation system. A CE mark was acquired. Using this kit, high-quality PRP can be prepared simply and inexpensively.^2^ Approximately 2 mL of PRP can be prepared from 20 mL of whole blood, and the platelet concentration rate is reportedly 7-fold.^2^ The growth factor content is also high.^2^ Clinically, the effects of PRP administration appear at a platelet count 4 to 7 times higher than the standard platelet count (200,000/μL).^15^ In addition, this kit facilitates the preparation of PRP using a versatile centrifuge that is used for clinical examinations; therefore, it is not necessary to purchase a special PRP-preparing instrument.

In the present case, PRP was prepared using the KYOCERA Medical PRP Kit, and wound healing of a fistula with subcutaneous cavity following bursitis of the lateral malleolus was successfully achieved. This is the first report on the use of this kit, which has produced the favorable course with no complications. In the future, the efficacy and usage of PRP should be examined in a randomized, controlled trial involving a large number of patients.

## References

[1] Kakudo N, Minakata T, Mitsui T, Kushida S, Notodihardjo FZ, Kusumoto K (2008). Proliferation-promoting effect of platelet-rich plasma on human adipose-derived stem cells and human dermal fibroblasts. *Plast Reconstr Surg*.

[2] Kushida S, Kakudo N, Morimoto N (2014). Platelet and growth factor concentrations in activated platelet-rich plasma: a comparison of seven commercial separation systems. *J Artif Organs*.

[3] Marx RE (2004). Platelet-rich plasma: evidence to support its use. *J Oral Maxillofac Surg*.

[4] Marx RE, Carlson ER, Eichstaedt RM, Schimmele SR, Strauss JE, Georgeff KR (1998). Platelet-rich plasma: growth factor enhancement for bone grafts. *Oral Surg Oral Med Oral Pathol Oral Radiol Endod*.

[5] Driver VR, Hanft J, Fylling CP, Beriou JM (2006). A prospective, randomized, controlled trial of autologous platelet-rich plasma gel for the treatment of diabetic foot ulcers. *Ostomy Wound Manage*.

[6] Willemsen JC, van der Lei B, Vermeulen KM, Stevens HP (2014). The effects of platelet-rich plasma on recovery time and aesthetic outcome in facial rejuvenation: preliminary retrospective observations. *Aesthetic Plast Surg*.

[7] Di Matteo B, Filardo G, Kon E, Marcacci M (2015). Platelet-rich plasma: evidence for the treatment of patellar and Achilles tendinopathy—a systematic review. *Musculoskelet Surg*.

[8] Filardo G, Kon E, Di Matteo B (2014). Platelet-rich plasma injections for the treatment of refractory Achilles tendinopathy: results at 4 years. *Blood Transfus*.

[9] Kakudo N, Kushida S, Kusumoto K (2009). Platelet-rich plasma: the importance of platelet separation and concentration. *Plast Reconstr Surg*.

[10] Kakudo N, Kushida S, Minakata T, Suzuki K, Kusumoto K (2011). Platelet-rich plasma promotes epithelialization and angiogenesis in a split-thickness skin graft donor site. *Med Mol Morphol*.

[11] Foster TE, Puskas BL, Mandelbaum BR, Gerhardt MB, Rodeo SA (2009). Platelet-rich plasma: from basic science to clinical applications. *Am J Sports Med*.

[12] Kakudo N, Morimoto N, Kushida S, Ogawa T, Kusumoto K (2014). Platelet-rich plasma releasate promotes angiogenesis in vitro and in vivo. *Med Mol Morphol*.

[13] Kushida S, Kakudo N, Suzuki K, Kusumoto K (2013). Effects of platelet-rich plasma on proliferation and myofibroblastic differentiation in human dermal fibroblasts. *Ann Plast Surg*.

[14] Krupski WC, Reilly LM, Perez S, Moss KM, Crombleholme PA, Rapp JH (1991). A prospective randomized trial of autologous platelet-derived wound healing factors for treatment of chronic nonhealing wounds: a preliminary report. *J Vasc Surg*.

[15] Marx RE, Garg AK (2005). Dental and Craniofacial Applications of Platelet-Rich Plasma.

